# The spatial and temporal situation of China’s digital technology innovation and its influencing factors

**DOI:** 10.1371/journal.pone.0297401

**Published:** 2024-01-29

**Authors:** Xiaoyu Wan, Yufan Wang, Wei Zhang

**Affiliations:** School of Economics and Management, Chongqing University of Posts and Telecommunications, Chongqing, China; Hosei University: Hosei Daigaku, JAPAN

## Abstract

Digital technology innovation is the core driving force for the high-quality development of the digital economy, and in-depth exploration of the regional distribution pattern and formation mechanism of digital technology innovation in China is conducive to the rational layout and coordinated development of the inter-provincial digital economy. Based on the Reference Relationship Table of the Classification of Core Industries of Digital Economy and the International Patent Classification (2023), the patent authorization data of digital technology from 2012 to 2022 were obtained, and the spatiotemporal situation of China’s digital technology innovation was analyzed by using ArcGIS software, Dagum’s Gini coefficient, and Moran’s I index, and the spatial Dubin panel model was used to explore the influencing factors of digital technology innovation. It is found that: (1) the scale and vitality of China’s digital technology innovation have increased significantly, and there are obvious spatial differentiation characteristics, and the innovation level of "eastern coastal—central and western interior" is decreasing, forming a cluster distribution pattern in the Yangtze River Delta region, Beijing, Guangdong, and other places, and the degree of agglomeration is decreasing. (2) The overall regional differences in China’s digital technology innovation are large, the differences between the East and the West dominate the interregional differences, and the net differences between regions are the main factors leading to regional differences. (3) There is a significant positive spatial correlation between the scale and vitality of digital technology innovation, which has a significant spatial spillover effect. (4) The results confirm that the level of economic development, digital access, financial scientific and technological support, technology market development level, and R&D intensity have a significant positive impact on the scale and vitality of digital technology innovation; The investment in scientific and technological talents has a significant positive impact on the scale of digital technology innovation, but has no significant impact on the vitality of digital technology innovation.

## 1. Introduction

At present, the digital economy has become an important starting point for China’s economic recovery. Since the 18th National Congress of the Communist Party of China, the Party Central Committee has attached great importance to the development of the digital economy and elevated it to a national strategy. The 19th National Congress of the Communist Party of China proposed to promote the deep integration of the Internet, big data, artificial intelligence, and the real economy to build a digital China and a smart society. The Fifth Plenary Session of the 19th CPC Central Committee proposed to develop the digital economy, promote digital industrialization and industrial digitalization, promote the deep integration of the digital economy and the real economy, and create a digital industrial cluster with international competitiveness. The 20th National Congress of the Communist Party of China proposed to insist on focusing on the real economy for economic development, promote new industrialization, and accelerate the construction of a manufacturing power, a quality power, a space power, a transportation power, a network power, and a digital China. The role of the digital economy in stabilizing growth and promoting transformation has become increasingly prominent, and it is an important force in changing China’s economic structure. According to the Digital China Development Report (2022) released by the Cyberspace Administration of China, the scale of China’s digital economy will exceed 50 trillion yuan for the first time in 2022, reaching 50.2 trillion yuan, accounting for 41.5% of GDP, and the scale of the digital economy has ranked second in the world for many consecutive years.

The digital economy has both economic and technical attributes [1], and the techno-economic paradigm behind it needs to be stripped out to maintain the rapid and stable growth of the digital economy [2]. Since the concept of the digital economy was proposed by Tapscott 1996 [3], authoritative organizations at home and abroad such as OEDC, BEM, China National Bureau of Statistics, China Academy of Information and Communications Technology, as well as domestic and foreign scholars such as Bukht R (2017) and Xu Xianchun (2020), have limited its definition to a technical framework [4,5], and the digital economy is regarded as a new economic form empowered by digital technology innovation [6,7]. From the perspective of technology, digital technology innovation is regarded as a disruptive innovation [8], unlike traditional technological innovation that only promotes or spawns technology-related industries, digital technology innovation not only gives birth to emerging industries—digital industries but also highly integrates with traditional industries to accelerate the process of digital transformation of traditional industries [6]. Digital technology is currently a general-purpose technology, and its participation in economic and social development is increasing day by day [9], which is not only the mainstay of a new round of scientific and technological revolution and industrial transformation but also changes the interaction mode and transaction channels of social participants [10]. It is also an important driving force for structural changes in social production relations, promoting a paradigm shift in techno-economic [11]. Driving the development of emerging industries and empowering the digital transformation of traditional industries with the help of digital technology innovation is an important choice to achieve the high-quality development of China’s digital economy [12,13]. Although China’s digital technology innovation has shown a good upward trend, the overall level is still low, and there is still a lot of room for improvement in the level of coordinated development with the digital economy [14,15].

Based on this, exploring the temporal and spatial situation and influencing factors of China’s digital technology innovation is necessary and urgent. Schumpeter regarded innovation as the main driving force for economic growth and development, and scientific and technological innovation not only had a pulling effect on the regional economy [16], and even dominated the development of the regional economy [17], but also had a spatial effect dominated by agglomeration effect and knowledge spillover effect, affecting scientific and technological innovation and economic development in neighboring regions [18,19]. Digital technology innovation covers scientific and technological innovation. The existing research on scientific and technological innovation and technological innovation has been relatively in-depth. However, the research on digital technology innovation is mainly based on qualitative research such as conceptual connotation, typical characteristics, and basic types [20–23]. Only a small number of scholars have proved the pulling effect and spatial effect of digital technology innovation on the economy [24,25], and there are still some research gaps: first, there are few quantitative studies on digital technology innovation, and the accuracy and objectivity of digital technology innovation level measurement need to be improved. Second, there is little research on digital technology innovation at the provincial level, and the spatial effect and driving mechanism between provinces need to be empirically tested.

Therefore, this paper attempts to define digital technology innovation as relevant technological innovation in the core industries of the digital economy, that is, the process of generating new technologies, new products, and new services through the reorganization of digital products or physical components of digital products or services [20], guided by the Reference Relationship Table of Classification of Core Industries in the Digital Economy and the International Patent Classification issued by the State Intellectual Property Office and the Statistical Classification of Digital Economy and its Core Industries (2021). Explore digital technology innovation’s spatial and temporal situation and its formation mechanism. On the one hand, through the IPC classification number and keywords of the core industries of the digital economy, the patent authorization data of 30 provincial-level administrative regions in China (except Hong Kong, Macao, Taiwan, and Xinjiang) from 2012 to 2022 were obtained. The spatial and temporal situation of China’s digital technology innovation was analyzed by using ArcGIS software, Dagum’s Gini coefficient, and Moran’s I index analysis methods. On the other hand, from the theoretical side, the possible influencing factors of provincial digital technology innovation are analyzed, and a spatial panel model is constructed to empirically test the possible influencing factors. To enrich the quantitative research of digital technology innovation and grasp the obstacles to the high-quality development of digital technology and the digital economy in China.

## 2. China’s digital technology innovation time and space bureau

### 2.1 Variable selection and data sources

#### 2.1.1 Variable selection

At present, there are two main ways to measure innovation. One is to build an innovation evaluation index system, through the analysis of innovation factor input, innovation factor output, innovation environment, innovation support, and many other influencing factors, find the main factors affecting the level of innovation. And empower and calculate the innovation index according to the degree of importance [26,27]. The second is to directly use patent data to measure the level of innovation. The patent is a widely recognized innovation measurement index, and the number of patent applications and authorizations in a certain field can objectively reflect the level of innovation in this field [25,28,29]. Compared with the construction of innovation evaluation indicators, the completeness and availability of patent data are recognized by many scholars. This paper selects the number of authorized invention patents and new utility patents to measure the level of digital technology innovation, and the results will be more accurate and objective [30]. To reflect the level of digital technology innovation in various provinces and cities more accurately, this paper decomposes the level of digital technology innovation into the scale of digital technology innovation (DI) and the vitality of digital technology innovation (PDI). Measuring DI by the number of patents authorized in the core industries of the digital economy, and the number of patent authorizations in the core industries of the digital economy by tens of millions of people.

#### 2.1.2 Data sources

The main source of patent data for digital technology innovation in this paper is the Dawei Patent Database. According to the "Reference Table of the Classification of Core Industries of the Digital Economy and the International Patent Classification (2023)" issued by the Office of the State Intellectual Property Office, the international patent classification symbols corresponding to digital technologies are counted, but not all patents belonging to the patent number are digital technology patents, and it is necessary to cooperate with the "Statistical Classification of Digital Economy and Its Core Industries (2021)" issued by the National Bureau of Statistics to screen digital technology patents and conduct an advanced search in Dawei patent database in the form of "international patent number + keywords". Obtained digital technology patent authorization data from 30 provinces and cities from 2012 to 2022.

### 2.2 Spatial distribution pattern of digital technology innovation

Selecting the two-time nodes of 2012 and 2022, the number of digital technology patent authorizations in 30 provincial administrative regions and the number of digital technology patent authorizations of tens of millions of people in China are divided into five levels through the natural fracture method of ArcGIS software, which can more intuitively present the relative pattern and change characteristics of digital technology innovation in China. The results show that China’s digital technology innovation shows a spatial pattern of decreasing innovation level of the "eastern coast-central and western interior". Compared with 2012, the level of digital technology innovation in 2022 will show a multiplied increase, and the polarization effect of the head provinces and cities will be more significant. From 2012 to 2022, from the perspective of the scale of digital technology innovation, Guangdong Province ranked first in the first echelon with a huge advantage, and the number of digital technology patents authorized far exceeded that of other provinces and cities. From the perspective of the vitality of digital technology innovation, Beijing and Shanghai are in the first echelon. As the political and economic centers of China, the two places lead the country in digital technology innovation vitality.

### 2.3 Analysis of regional differences in digital technology innovation

#### 2.3.1 Regional differences in space-time

**Fig 1:**
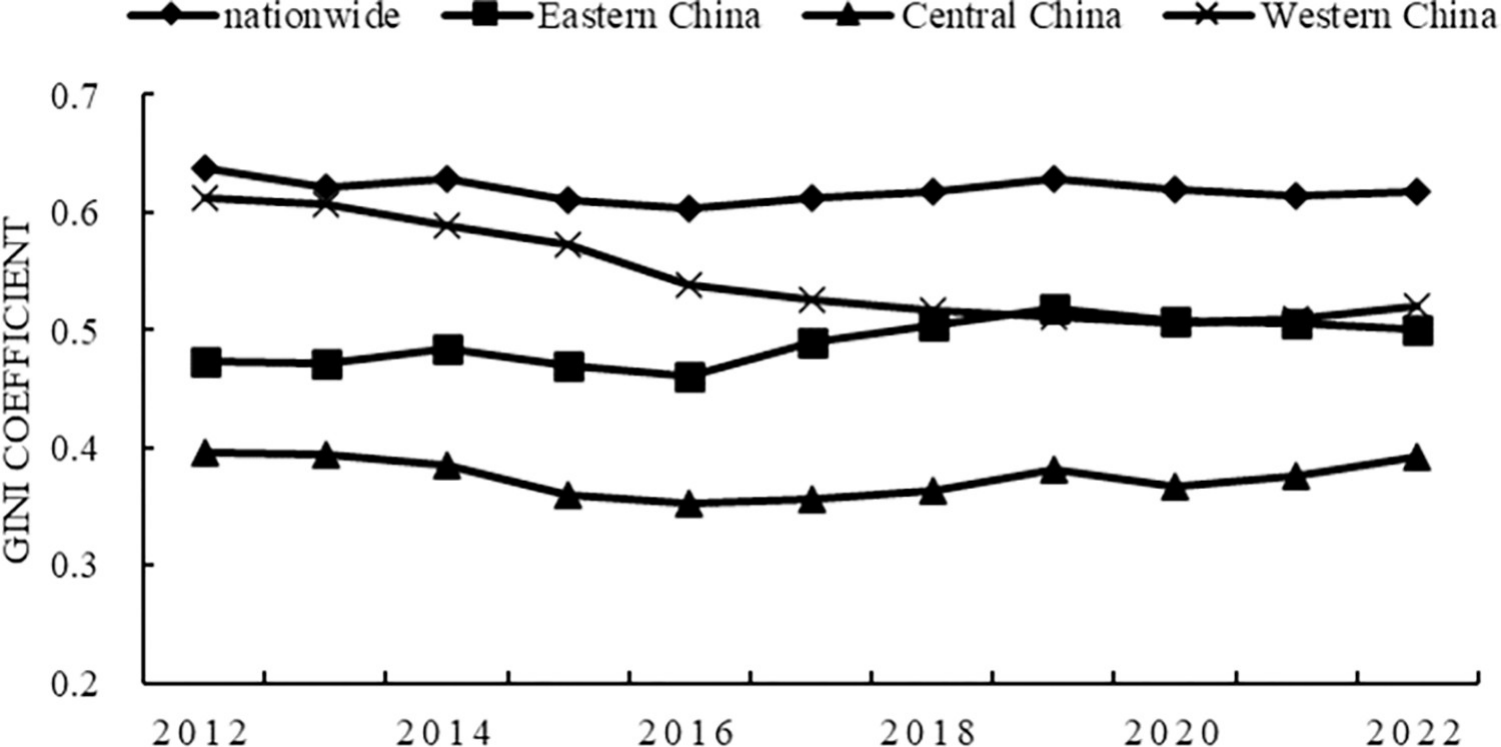
Gini coefficient of digital technology innovation scale.

**Fig 2:**
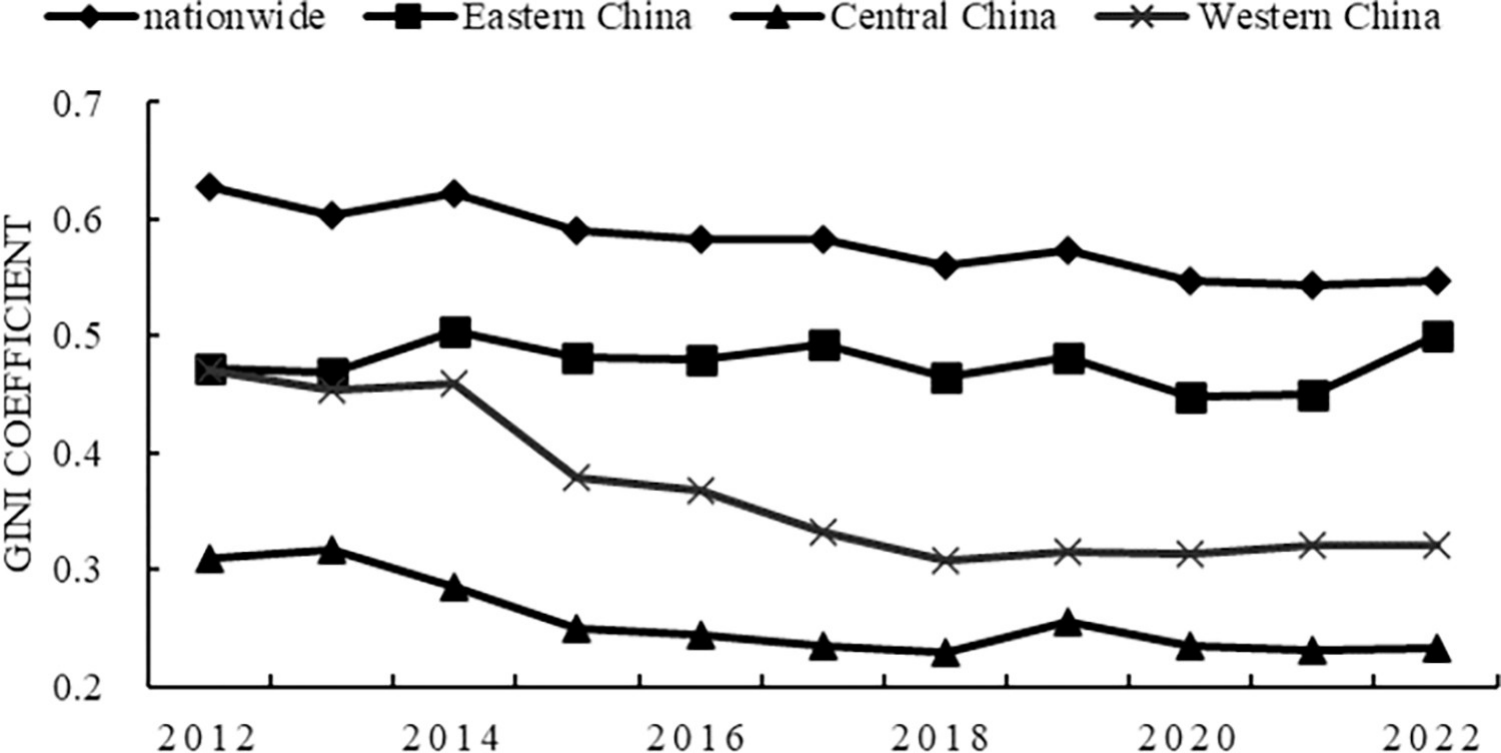
Gini coefficient of the vitality of digital technology innovation.

Dagum’s Gini coefficient was used to analyze and decompose the spatiotemporal differences. Figs 1 and 2 report the overall differences in the scale and vitality of China’s provincial digital technology innovation from 2012 to 2022, and the evolution trend of regional differences. From the average point of view, the regional differences in the scale of digital technology innovation are from high to low, in order of western region, eastern region, and central region. The differences within regions of digital technology innovation vitality are in descending order from the eastern, western, and central regions. The basic characteristics of the difference between the two are as follows: first, the national difference level is the highest, showing a slow downward trend during the observation period, and will maintain a large difference level in the future. Second, the central region has the lowest level of difference, showing a downward trend during the observation period, and the central region difference will remain low for a long time in the future. Third, the western region converged the fastest. The scale of digital technology innovation and the vitality Gini coefficient of digital technology innovation decreased by 14.88% and 31.79% respectively, and the regional differences continued to narrow rapidly. Fourth, as of 2022, the Gini coefficient in the eastern region is the highest among regions, and the Gini coefficient of digital technology innovation scale shows a clear upward trend, and the regional differences are gradually expanding.

#### 2.3.2 Difference decomposition

Dagum breaks down the Gini coefficient into three parts, namely, the contribution of within-subgroup differences to the overall difference, the contribution of net differences between subgroups to the overall difference, and the contribution of super variable density between subgroups to the overall difference, which are collectively called the contribution of the intergroup differences to the overall difference. Tables 1 and 2 are the results of decomposing the overall differences in the scale and vitality of digital technology innovation into intra-group and between-group differences according to Dagum’s decomposition method. The between-group differences are further decomposed into net between-group differences and over-variable densities. The results show that the main source of difference in the scale and vitality of digital technology innovation is the net difference between groups, with an average contribution rate of 63.95% and 65.96%, respectively. This was followed by within-group differences and hypervariable density, with average contribution rates of 27.46% and 26.75%, 8.61% and 7.29%, respectively. It can be seen that there are great differences in the scale and vitality of digital technology innovation in China. The main reason for the difference is the large gap in the level of digital technology innovation between the eastern, central, and western regions. Second, there are gaps in the level of digital technology innovation between provinces and cities within the region. Regional differences are also an important factor in the overall variation. From the perspective of evolutionary trends, the scale and vitality of digital technology innovation show a similar trend. That is, the fluctuation of intra-group differences increases, the net difference between groups and the fluctuation of super variable density decreases, that is, the gap between provinces and cities within the region is widening, and the gap between regions is narrowing.

**Table 1:** Breakdown of overall differences in the scale of digital technology innovation.

year	Within-subgroup differences		Between-subgroup differences			
			Net between-subgroup differences		Supervariable density	
	contribute	Contribution rate(%)	contribute	Contribution rate (%)	contribute	Contribution rate(%)
2012 2013 2014 2015 2016 2017 2018 2019 2020 2021 2022	0.1691 0.1686 0.1710 0.1652 0.1610 0.1685 0.1721 0.1771 0.1732 0.1727 0.1725	26.5197 27.0895 27.1968 27.0541 26.7059 27.4920 27.8768 28.1708 27.9421 28.0923 27.9014	0.4168 0.3897 0.3969 0.3870 0.3907 0.3916 0.3935 0.4019 0.4007 0.3929 0.3940	65.3798 62.6185 63.1236 63.3895 64.8009 63.9063 63.7442 63.9435 64.6346 63.9114 63.7469	0.0516 0.0640 0.0609 0.0583 0.0512 0.0527 0.0517 0.0496 0.0460 0.0492 0.0516	8.1006 10.2919 9.6796 9.5565 8.4931 8.6017 8.3790 7.8857 7.4234 7.9964 8.3517

**Table 2:** Breakdown of overall differences in the vitality of digital technology innovation.

year	Within-subgroup differences		Between-subgroup differences			
			Net between-subgroup differences		Supervariable density	
	contribute	Contribution rate(%)	contribute	Contribution rate(%)	contribute	Contribution rate (%)
2012 2013 2014 2015 2016 2017 2018 2019 2020 2021 2022	0.1626 0.1603 0.1696 0.1576 0.1562 0.1578 0.1481 0.1544 0.1434 0.1439 0.1484	25.9288 26.5514 27.2773 26.6871 26.7716 27.1093 26.3982 26.9069 26.1995 26.5002 27.6736	0.4233 0.3904 0.4017 0.3872 0.3848 0.3802 0.3724 0.3821 0.3721 0.3657 0.3732	67.4980 64.6476 64.5938 65.5603 65.9477 65.3139 66.3758 66.5985 67.9716 67.3223 67.0432	0.0412 0.0532 0.0506 0.0458 0.0425 0.0441 0.0405 0.0373 0.0319 0.0336 0.0350	6.5732 8.8010 8.1289 7.7526 7.2807 7.5768 7.2261 6.4946 5.8289 6.1775 6.2831

### 2.4 Digital technology innovation spatial autocorrelation

This paper uses Moran’s I index to test spatial autocorrelation. The global Moran’s I index is commonly used to measure the overall spatial association characteristics of all study subjects within a spatial range. It can scientifically react to the agglomeration characteristics of elements in the study space. The value range is between -1 and 1, and the closer to 1, the stronger the positive correlation between provinces. The closer to -1, the stronger the negative correlation between provinces. Proximity to 0 indicates that there is no spatial autocorrelation between provinces. Local Moran’s I index characterizes the agglomeration of local space. Under the premise of global autocorrelation space, it can reveal the spatial heterogeneity of the distribution of features centered on a single spatial unit, and more delicately depict the spatial agglomeration characteristics of features. If the local Moran’s I index is significant, a single spatial unit can be divided into four agglomeration forms: high-high, high-low, low-high, and low-low through the standard values of a single spatial unit variable.

As shown in Tables 3 and 4, under the setting of adjacency spatial weight matrix and inverse distance spatial weight matrix, the scale of provincial-level digital technology innovation and vitality spatial autocorrelation in China from 2012 to 2022 is significantly positive at a significant level of 1%. There is a significant positive correlation. The global Moran’s I index value fluctuated and increased during the observation period. Nationwide, the agglomeration characteristics of digital technology innovation level are gradually strengthening. Further, the partial Moran’s I index test of the vitality of digital technology innovation shows that (Table 5) the scale and vitality of China’s digital technology innovation are high-high, high-low, low-high, and low-low spatial aggregation types. However, it is mainly a high-high and low-low agglomeration state. Taking 2012 and 2022 as examples, the high-high aggregation of the scale and vitality of digital technology innovation is mainly concentrated in the Yangtze River Delta region. The high-scale and high-aggregation state of digital technology innovation specifically includes Jiangsu, Shanghai, Zhejiang, Fujian, and Anhui. The state of high vitality and high aggregation of digital technology innovation specifically includes Jiangsu, Shanghai, and Zhejiang. It shows that the Yangtze River Delta region is the most prominent agglomeration area with the most prominent level of digital technology innovation in China. Although the level of digital technology innovation in Guangdong, Beijing, and other places is also prominent, the level of digital technology innovation in neighboring provinces and cities is low, and high-high agglomeration areas have not been formed. Low-low spatial agglomeration types were mainly concentrated in Xinjiang, Gansu, and Inner Mongolia in Northwest China. Compared with 2012, the scale of digital technology innovation and the distribution of low-vitality-low spatial agglomeration types in 2022 have changed, and Inner Mongolia has changed from an insignificant state in 2012 to a low-low agglomeration state. In 2022, Gansu will get rid of the low-low agglomeration state of digital technology innovation. Except for Sichuan, there are no significant high-low agglomeration areas, and Sichuan will be out of the high-low agglomeration state of digital technology innovation vitality in 2022. Except for Jiangxi, the scale of digital technology innovation has no significant low-high agglomeration areas. The vitality of digital technology innovation does not have a low-high agglomeration area.

**Table 3:** Global Moran Index on the scale of digital technology innovation.

year	Adjacency spatial weights matrix		Inverse distance space weights matrix	
	Moran’s I index	P-value	Moran’s I index	P-value
2012 2013 2014 2015 2016 2017 2018 2019 2020 2021 2022	0.3525 0.3504 0.3617 0.3826 0.4032 0.3778 0.4124 0.4204 0.4342 0.4151 0.4143	0.0014 0.0016 0.0011 0.0006 0.0003 0.0007 0.0002 0.0002 0.0001 0.0002 0.0002	0.2770 0.2584 0.2769 0.2633 0.2769 0.2397 0.2654 0.2760 0.2915 0.2839 0.2646	0.0002 0.0005 0.0002 0.0004 0.0002 0.0011 0.0004 0.0002 0.0001 0.0002 0.0002

**Table 4:** Global Moran Index of Digital Technology Innovation Vitality.

year	Adjacency spatial weights matrix		Inverse distance space weights matrix	
	Moran’s I index	P-value	Moran’s I index	P-value
2012 2013 2014 2015 2016 2017 2018 2019 2020 2021 2022	0.3830 0.3672 0.3906 0.3998 0.4195 0.3761 0.4194 0.4098 0.4321 0.4179 0.4057	0.0006 0.0010 0.0005 0.0003 0.0002 0.0007 0.0002 0.0002 0.0001 0.0002 0.0002	0.2616 0.2459 0.2023 0.2476 0.2812 0.2421 0.2541 0.2090 0.2357 0.2405 0.2033	0.0042 0.0041 0.0070 0.0019 0.0008 0.0026 0.0032 0.0096 0.0061 0.0047 0.0098

**Table 5:** Global autocorrelation of the scale and dynamism of digital technology innovation.

Digital technology innovation scale			Digital technology innovation dynamism	
Agglomeration type	2012	2022	2012年	2022年
High-high	Jiangsu, Shanghai, Zhejiang, Fujian, Anhui	Jiangsu, Shanghai, Zhejiang, Fujian, Anhui	Jiangsu, Shanghai, Zhejiang	Jiangsu, Shanghai, Zhejiang
High-low	Sichuan	Sichuan	Sichuan	-
Low-high	Jiangxi	Jiangxi	-	-
Low-low	Xinjiang, Gansu	Xinjiang, Gansu, Inner Mongolia	Xinjiang, Gansu	Xinjiang, Inner Mongolia
Not significant	other	other	other	other

## 3. Testing of influencing factors of digital technology innovation

### 3.1 Variable selection and data sources

The selection of explanatory variables and data sources in this paper have been discussed earlier.

#### 3.1.1 Explanatory variable selection

By combing the relevant literature on digital technology innovation, it is found that the influencing factors of digital technology innovation can be mainly summarized as digital technology innovation environment and digital technology innovation factor input [25,27,31,32]. This paper selects the level of economic development, digital access levels, financial support for science and technology, and technology market development as potential influencing factors on the environmental side of digital technology innovation. R&D intensity and investment in scientific and technological talents were selected as potential influencing factors on the input side of digital technology innovation to explore.

The definitions of relevant variables in this paper are shown in Table 6.

**Table 6:** Explanatory variable definition.

Variable	Symbol	Measure
Level of economic development	*ED*	Regional GDP / Total population of the region
Level of digital access levels	*DA*	Number of Internet broadband access ports
Level of financial support for science and technology	*FS*	Fiscal expenditure on science and technology/regional GDP
Level of technology market development	*TMD*	Technology market turnover / regional GDP
R&D intensity	*RDI*	Internal expenditure on R&D funding/regional GDP
Investment in scientific and technological talents	*STT*	Full-time equivalent of R&D personnel

#### 3.1.2 Explanatory variable data source

Per capita GDP, number of Internet broadband access ports, proportion of local financial science and technology expenditure to GDP, proportion of technology market turnover to GDP, full-time equivalent of R&D personnel, and proportion of internal expenditure of R&D funds to GDP are mainly derived from China Statistical Yearbook, Local Statistical Yearbook, Science and Technology Statistical Yearbook, Mark Data Network, etc. Due to the lack of a large number of statistics for 2022, all data is as of 2021. Missing data from 2012 to 2021 were completed by interpolation. And take the natural logarithm treatment of all variables to reduce the impact of too many orders of magnitude differences between different variables.

### 3.2 Metrological model selection

The visual representation of the ArcGIS software and the results of Moran’s I Index show that digital technology innovation has a clear spatial dependence. Therefore, this paper constructs a spatial panel model to explore the influencing factors and spatial spillover effects of China’s provincial digital technology innovation level. The three common forms of spatial panel models are the spatial autoregressive model (SAR), spatial error model (SEM), and spatial Dubman model (SDM). Among them, the spatial Dubin model adds both the spatial lag term of the explanatory variable and the spatial lag term of the explanatory variable, and the model is as follows:

$${PDI}_{it}=\alpha_{0}+\beta X_{it}+\rho W{PDI}_{it}+\theta WX_{it}+\mu_{i}+v_{t}+\varepsilon_{it}$$ (1)

In Eq (1), W represents the spatial weights matrix; ρWPDI_it_ represents the spatial lag term of the vitality of digital technology innovation; WX_it_ spatial lag term represents the influencing factor; β and θ represent the parameters to be estimated for the space lag term; μ_i_ and v_t_ indicate individual effects and time effects; ε_it_ represents a random perturbation term. The spatial Dubin model can simultaneously investigate the impact of the influencing factors of digital technology innovation in neighboring regions on digital technology innovation in the region, and decompose the total utility into direct effects and indirect effects, which are spatial spillover effects.

### 3.3 Selection test of a spatial econometric model

According to the calculation results of the global Moran’s I index of the scale and vitality of digital technology innovation, it can be seen that there is a spatial autocorrelation in the scale and vitality of digital technology innovation in China. Therefore, the analysis of its influencing factors needs to take into account the spatial spillover effect. A spatial weight matrix is introduced to estimate the spatial econometric model. Drawing on the practice of J.P. Elhorst, OLS regression is carried out first, and the Lagrange (LM) test is used to determine whether there is a spatial lag effect and a spatial error effect. If present, select the spatial panel model. After passing the LM test, the Hausman test is performed to determine whether the spatial regression model is suitable for fixed effects or random effects. Then the likelihood ratio (LR) test and the Wald test are carried out to determine whether the spatial Dubin model will degenerate into a spatial lag model or a spatial error model.

The results (Table 7) show that the scale of digital technology innovation has passed the LM test of spatial error effect, Robust-LM test, and Robust-LM test of spatial lag effect at the significance level of 1%, and failed the LM test of spatial lag effect. The vitality of digital technology innovation has passed the LM test of spatial error effect at the significance level of 1%, the Robust-LM test and the LM test of spatial lag effect at the significance level of 1%, and the Robust-LM test of the spatial lag effect at the significance level of 10%. Therefore, the spatial Dubin model is preliminarily selected, and which spatial panel model is more suitable to use is further judged according to the subsequent test. The Hausman test for the scale and dynamism of digital technology innovation rejected the null hypothesis at a significance level of 10% and 1%, respectively, so the fixed-effect model was chosen. The LR test and Wald test of the scale and vitality of digital technology innovation are significant at the significance level of 1%, except for the Wald test of the spatial error model of digital technology innovation scale, which is only significant at the significance level of 5%. The SDM model that illustrates the scale and vitality of digital technology innovation can degenerate into the SAR model and SEM model. Therefore, the fixed-effect spatial Durman model is chosen in this paper. Further regression analysis of the SDM model shows that the scale and vitality of digital technology innovation are R^2^ = 0.9245 and R^2^ = 0.8960 under the temporal fixed effect. All of them were higher than the R^2^ values under individual fixed and double fixed effects, so the spatial Dubin model with selected temporal fixed effects had the highest goodness of fit.

**Table 7:** Model test results.

	Digital technology innovation scale (DI)		Digital technology innovation dynamism (PDI)	
Inspection method	Regression coefficient	P-value	Regression coefficient	P-value
LM error	59.433	0.000	15.935	0.000
Robust LM error	67,189	0.000	8.709	0.000
LM lag	0.596	0.440	10.660	0.003
Robust LM lag	8.352	0.004	3.434	0.064
Hausman	20.25	0.089	29.07	0.006
LR Lag	53.49	0.000	54.21	0.000
LR Err	53.67	0.000	54.38	0.000
Wald Lag	22.69	0.001	20.33	0.001
Wald Err	13.62	0.034	16.80	0.005

### 3.4 Analysis of estimated results

Columns (1) and (4) in Table 8 report the estimation of the influencing factors of the scale and vitality of China’s digital technology innovation under the setting of spatial adjacency matrix from 2012 to 2021. Based on the empirical results, the following analysis was carried out.

**Table 8:** Spatial Dubin panel model estimation results.

	Digital technology innovation scale			Digital technology innovation dynamism		
variable	(1)	(2)	(3)	(4)	(5)	(6)
lnPGDP	0.420*** (5.00)	0.365*** (3.69)	0.262*** (3.00)	1.276*** (15.22)	1.219*** (11.53)	1.045*** (11.85)
lnDA	0.676*** (8.57)	0.654*** (7.74)	0.743*** (8.76)	0.220*** (2.89)	0.240*** (2.67)	0.215** (2.50)
lnFS	0.130*** (2.64)	0.172*** (2.99)	0.191*** (3.74)	0.238*** (5.01)	0.289*** (4.74)	0.279*** (5.37)
lnTMD	0.056** (2.46)	0.063*** (2.83)	0.070*** (2.96)	0.050** (2.29)	0.074*** (3.11)	0.079*** (3.30)
lnSTR	0.585*** (5.68)	0.696*** (6.57)	0.743*** (7.01)	0.802*** (8.13)	0.859*** (7.65)	0.937*** (8.73)
lnSTT	0.393*** (5.37)	0.426*** (5.74)	0.345*** (4.65)	-0.071 (-1.00)	-0.074 (-0.93)	-0.084 (-1.11)
W×lnPGDP	0.701*** (3.64)	0.547 (0.63)	-0.362 (-1.51)	1.425*** (6.60)	1.935** (1.99)	-0.212 (-0.79)
W×lnDA	0.401** (2.15)	2.440*** (4.42)	0.537** (2.36)	0.459*** (2.74)	2.753*** (5.07)	0.813*** (3.88)
W×lnFS	0.176 (1.60)	-0.063 (-0.14)	0.098 (0.53)	0.007 (0.07)	-0.048 (-0.10)	0.057 (0.31)
W×lnTMD	-0.149*** (-3.45)	-0.067 (-0.40)	-0.074 (-1.51)	-0.112*** (-2.67)	0.420** (2.35)	-0.023 (-0.46)
W×lnSTR	0.946*** (4.55)	-0.418 (-0.59)	-0.282 (-1.05)	0.746*** (3.55)	-0.880 (-1.17)	-0.686** (-2.48)
W×lnSTT	-0.811*** (-5.22)	-1.413*** (-3.20)	-0.591*** (-3.22)	-0.845*** (-5.67)	-1.974*** (-4.22)	-0.681*** (-3.65)
rho	0.354*** (4.58)	0.251** (2.46)	0.254*** (2.58)	0.372*** (4.87)	0.251** (2.39)	0.272*** (2.87)
sigma2_e	0.076*** (12.22)	0.073*** (12.53)	0.083*** (12.07)	0.071*** (12.25)	0.082*** (12.35)	0.085*** (12.25)
Observations	300	300	300	300	300	300
R-squared	0.9245	0.8216	0.9575	0.8960	0.6497	0.9272

Note

*, **, **** indicate p<0.01, p<0.05, p<0.1, respectively, and the z value in parentheses.

#### 3.4.1 Digital technology innovation environment

The level of economic development (PGDP) and its spatial lag are both at a significant level of 1%, which has a positive impact on the scale and vitality of China’s digital technology innovation. It shows that the level of economic development has a local effect on the region and a diffusion effect on the neighboring areas. The level of economic development can largely reflect the comprehensive strength of the region. The elements of digital technology innovation tend to converge in regions with strong comprehensive strength. However, the carrying capacity of factors in a region is limited, and when the agglomeration of digital technology innovation factors in a certain place is too high, it will produce a diffusion effect. Factors will give priority to flowing to neighboring regions, driving the level of digital technology innovation in surrounding areas to improve [33]. Therefore, the level of economic development has a significant role in promoting digital technology innovation in the region and neighboring regions.

Digital access (DA) has a positive impact on the scale and vitality of digital technology innovation in China at a significant level of 1%. Its spatial lag items have a positive impact on the scale and vitality of China’s digital technology innovation at the significance level of 5% and 1%, respectively. It shows that digital access has a significant role in promoting the level of digital technology innovation in the region and neighboring regions. Digital access is an effective way to improve the efficiency of information dissemination. It can not only meet the infrastructure needs of digital technology innovation but also improve the connectivity of information networks within and between regions and the collaborative efficiency of digital technology innovation. In turn, it will increase the output of digital technology innovation and enhance the vitality of digital technology innovation.

Financial science and technology support (FS) has a positive impact on the scale and vitality of China’s digital technology innovation at a significant level of 1%. Its spatial lag term is not significant. It shows that financial scientific and technological support has a significant role in promoting digital technology innovation in the region, but the impact on neighboring regions is not significant. Financial scientific and technological support is the embodiment of the extent of regional financial support for its own scientific and technological activities. Digital technology innovation is a kind of subordinate to science and technology activities, and the greater the degree of financial support, the stronger the promotion effect on digital technology innovation in the region [34,35].

The Technology Market Development Level (TMD) has a positive impact on the scale and vitality of China’s digital technology innovation at a significant level of 5%. Its spatial lag term hurts the scale and vitality of China’s digital technology innovation at a significant level of 1%. It shows that the development level of the technology market has a significant role in promoting digital technology innovation in the region, and has a significant inhibitory effect on digital technology innovation in neighboring regions. Technology market turnover is an important indicator of a region’s ability to innovate and transform science and technology [36,37]. The continuous improvement of the development level of the technology market means that technology transactions are active and the innovation environment is optimized. This creates a siphon effect on elements of digital technology innovation. Accelerate the agglomeration of surrounding capital, talents, and other factors to regions with a higher level of technology market development. This will promote digital technology innovation in the region and suppress digital technology innovation in neighboring regions.

#### 3.4.2 Elements of digital technology innovation

R&D intensity (STR) and its spatial lag term are both at a significant level of 1%, which has a positive impact on the scale and vitality of China’s digital technology innovation. It shows that R&D intensity has a significant role in promoting the scale and scale of digital technology innovation in this region and neighboring regions. On the one hand, regions with high R&D intensity can provide better funding for their research of new theories, new products, and technologies. This ensures the continuity of R&D needs and material needs for R&D are fully met, thereby increasing the success rate of digital technology innovation in the region [38]. On the other hand, theory and technology have fluidity. Cutting-edge theories and advanced technologies will be preferentially diffused to surrounding areas [39], thereby promoting digital technology innovation in neighboring regions.

The investment in scientific and technological talents (STT) has a positive impact on the scale of digital technology innovation in China at a significant level of 1%. The impact on the vitality of China’s digital technology innovation is not significant. The coefficients of the spatial lag term for both are negative and significant at the significance level of 1%. It shows that the investment of scientific and technological talents has a significant role in promoting the scale of digital technology innovation in the region. The impact of the dynamism of digital technology innovation in the region is not obvious. It has a significant inhibitory effect on the scale and vitality of digital technology innovation in neighboring regions. Scientific and technological talents are the most active element in digital technology innovation activities. The flow of scientific and technological talents is affected by "excellence" and "profit-seeking", and will flow to regions with a larger scale of scientific and technological talents, and better realize their value through teamwork and other means [40]. Therefore, the scale of digital technology innovation will increase with the increase of investment in scientific and technological talents. Neighboring regions will lose some scientific and technological talents due to the siphon effect, thereby inhibiting digital technology innovation. The vitality of digital technology innovation is a per capita concept, so the absolute amount of investment in scientific and technological talents has no significant impact on the vitality of digital technology innovation in the region.

### 3.5 Robustness test

To strengthen the robustness of the experimental results, this paper constructs the inverse distance spatial weight matrix and the economic distance spatial weight matrix and performs a spatial panel regression analysis based on the spatial Dubin model of the influencing factors of digital technology innovation. As shown in Table 8, columns (2) and (3) are the estimation results of the influencing factors of digital technology innovation scale under the setting of the inverse distance spatial weight matrix and the economic distance spatial weight matrix. Columns (5) and (6) are the estimation results of the influencing factors of digital technology innovation vitality under the setting of the inverse distance spatial weight matrix and the economic distance spatial weight matrix. The impact of economic development level, digital access level, financial scientific and technological support, technology market development level, and R&D intensity on the scale and vitality of China’s digital technology innovation under the inverse distance matrix and inverse distance square matrix are significantly positive. Under these two matrices, the impact of scientific and technological talent investment on the scale of digital technology innovation is significantly positive, and the impact effect on the vitality of digital technology innovation is not significant. The results are highly consistent with the estimates under the adjacency matrix. Under the three spatial weight matrices, the influence of the spatial lag term coefficient supported by financial science and technology on the scale and vitality of digital technology innovation was not significant. The spatial lag term of the digital access level has a significantly positive effect on it. The spatial lag item of scientific and technological talent investment has a significantly negative impact on it. Estimates are consistent. There are certain differences in the estimation results of the spatial lag in terms of economic development level, technology market development level, and R&D intensity under the three spatial weight matrices. Its effect needs to be further tested. In general, the estimation results of the analysis of influencing factors of digital technology innovation are consistent under the three spatial weight matrices. The results show that the estimation results of the spatial Durman model based on fixed effects are robust and the analysis content is more reliable.

## 4. Conclusions and recommendations

### 4.1 Conclusion of the study

Digital technology innovation is an important starting point for achieving high-quality development of the digital economy. This paper analyzes the level of digital technology innovation in 30 provinces and cities in China from 2012 to 2021 through authorized patent data. ArcGIS software is used to visualize the temporal and spatial evolution pattern of digital technology innovation. On this basis, the Dugum Gini coefficient is used to calculate the difference in the level of digital technology innovation in the eastern, central, and western parts of China. and break down the sources of difference. The global Moran’s I index was used to analyze the spatial autocorrelation of digital technology innovation under different spatial weight matrices. Under the premise of strong global autocorrelation, the local Moran’s I index is used to further explore the spatial agglomeration characteristics. The SDM model was used to analyze the influencing factors of digital technology innovation. The conclusions are as follows:

First, from 2012 to 2022, the scale and vitality of China’s digital technology innovation have increased significantly. The number of digital technology patents granted by provinces and cities increased exponentially during the study period. The scale and vitality of China’s digital technology innovation are spatially manifested in a decreasing trend from eastern coastal provinces and cities to inland provinces and cities, and there are obvious differentiation characteristics. The results of Moran’s I indexshow that there is a significant spatial positive correlation between digital technology innovation in China. It is distributed in clusters in the Yangtze River Delta region. The northwestern inland provinces such as Xinjiang, Gansu, and Inner Mongolia are regions with relatively small scale and vitality in digital technology innovation.

Second, it can be known from the Dugum Gini coefficientand its decomposition results. The scale of China’s digital technology innovation converges the fastest in the western region. The central region remained stable with minimal differences. Disparities in the eastern region are widening. China’s digital technology innovation vitality, the western and central regions have shown a clear convergence trend. The central difference is minimal. The eastern region remained stable and had the greatest variation. The net difference between groups is the most important factor leading to the regional difference in the scale and vitality of digital technology innovation in China. Its average contribution rate is more than 60%.

Third, the regression results of the SDM model under different spatial weight matrices show that the level of economic development, digital access, financial and technological support, technology market development level, and R&D intensity have a significant positive impact on digital technology innovation in the region. The investment in technology talents has a significant positive impact on the scale of local digital technology innovation. It has no significant impact on the vitality of digital technology innovation. Financial and technological support has no significant impact on the scale and vitality of digital technology innovation in neighboring regions. The level of digital access has a significant positive impact on the scale and vitality of digital technology innovation in neighboring regions. The investment of scientific and technological talents has a significant negative impact on the scale and vitality of digital technology innovation in neighboring regions. The influence of economic development level, technology market development level, and R&D intensity on neighboring regions needs to be further tested.

### 4.2 Policy recommendations

Digital technology innovation is the core driving force for the high-quality development of the digital economy and an important way for the high-quality development of the regional economy. Government departments should fully consider the spatial effects and impact mechanisms of digital technology innovation when making top-level designs. Give full play to the role of the two hands of the government and the market to maximize the value of each input factor.

Based on the empirical analysis of this paper, the following policy recommendations are proposed:

First, strengthen top-level design at the national level to promote balanced and coordinated development among regions. Adhere to the idea of "one game of chess for the whole country", and reduce or even break down barriers to the flow of innovation factors between provinces by providing financial subsidies, unifying market access systems, and adjusting tax rates. Promote the sharing and interoperability of infrastructure, data resources, and human resources between regions, and strengthen the linkage between provinces and cities [41]. Clarify the resource endowments of different provinces and cities for the development of the digital economy. By increasing forms such as central-to-local transfer payments, resources will be tilted towards regions with low digital technology innovation capabilities. Guide provinces and cities to find the correct development positioning and give play to their advantages. Strive to form a pattern of digital technology innovation and digital economy development with distinctive characteristics and outstanding competitiveness in each region nationwide.

Second, actively create a good environment for innovation. On the one hand, local governments should rationally deploy and build new infrastructure in 5G base stations, big data centers, artificial intelligence, and other fields. Relying on the region’s factor endowments and industrial advantages, build a digital basic platform. Provide hard environment guarantee for digital technology innovation activities. On the other hand, local governments should increase investment in science and technology, accelerate the development of technology markets, improve technology transfer mechanisms, encourage enterprises to strengthen investment in research and development, and introduce targeted talent policies and other measures to create a scientific and technological innovation atmosphere that respects knowledge, creativity and talents. Provide soft environment support for digital technology innovation activities.

Third, actively guide enterprises to assume the responsibility of innovation subjects. From financial subsidies, tax incentives, financial support, talent support, technology market environment optimization, and other points. Encourage local enterprises, especially enterprises with local characteristics and leading enterprises, to carry out digital technology innovation activities. Guided by the demand for digital technology in the development of the digital economy, highlight the core position of basic research in innovation activities. Fully stimulate the innovation vitality of the enterprise side. While increasing innovation output, accelerate the transformation of innovation results.

Fourth, scientifically build a university training system for scientific and technological talents, and actively introduce policies for scientific and technological talents. According to the current development level and trend of the digital economy and digital technology, forward-looking deployment and construction of related majors in colleges and universities. Give full play to the role of colleges and universities as the main position of talent training, and provide strong talent support and talent reserves for digital technology innovation. Scientific and technological talents are a key part of regional scientific and technological activities and the construction of regional innovation systems [42]. It is necessary to give full play to regional advantages and take multiple measures in the "attraction, retention, and transfer" of scientific and technological talents. Accelerate the flow and agglomeration of talent elements and build a solid foundation for digital technology innovation.

## Supporting information

S1 Dataset (XLSX)
